# Diagnosis and Anti-Reflux Therapy for GERD with Respiratory Symptoms: A Study Using Multichannel Intraluminal Impedance-pH Monitoring

**DOI:** 10.1371/journal.pone.0160139

**Published:** 2016-08-17

**Authors:** Chao Zhang, Jimin Wu, Zhiwei Hu, Chao Yan, Xiang Gao, Weitao Liang, Diangang Liu, Fei Li, Zhonggao Wang

**Affiliations:** 1 Department of General Surgery, Xuanwu Hospital, Capital Medical University, No. 45 Changchun Street, Xicheng District, Beijing, 100053, China; 2 Department of Gastroesophageal Reflux Disease, Second Artillery General Hospital of Chinese People’s Liberation Army, No.16 Xinjiekou Street, Xicheng District, Beijing, 100088, China; National Cancer Center, JAPAN

## Abstract

**Background/Aims:**

Respiratory symptoms are often associated with gastroesophageal reflux disease (GERD). Although the role of multichannel intraluminal impedance–pH (MII-pH) monitoring in GERD is clear, little is known regarding the characteristics of patients with respiratory symptoms based on MII-pH monitoring and anti-reflux therapy. We evaluated a cohort of GERD patients to identify the MII-pH parameters of GERD-related respiratory symptoms and to assess the anti-reflux therapy outcomes.

**Methods:**

We undertook a prospective study of patients who were referred for GERD evaluation from January 2011 to January 2012. One hundred ninety-five patients underwent MII-pH monitoring and esophageal manometry, and one hundred sixty-five patients underwent invasive anti-reflux therapy that included laparoscopic Toupet fundoplication (LTF) and the Stretta procedure. The patient characteristics and MII-pH parameters were analyzed, and the symptom scores were assessed at baseline and at 1- and 3-year follow-up evaluations.

**Results:**

Of the 195 patients, 96 (49.2%) exhibited respiratory symptoms and significantly more reflux episodes (70.7±29.3) than patients without respiratory symptoms (64.7±24.4, *p* = 0.044) based on the MII-pH monitoring results. Moreover, the group of patients with respiratory symptoms exhibited more proximal reflux episodes (35.2±21.3) than the non-respiratory symptomatic group (28.3±17.9, *p* = 0.013). One hundred twenty-five patients following the Stretta procedure (n = 60, 31 with respiratory symptoms) or LTF (n = 65, 35 with respiratory symptoms) completed the designated 3-year follow-up period and were included in the final analysis. The symptom scores after anti-reflux therapy all decreased relative to the corresponding baseline values (*p*<0.05), and there were no significant differences in the control of respiration between the Stretta procedure and LTF (*p*>0.05). However, LTF significantly reduced the recurrence (re-operation) rate compared with the Stretta procedure (0 vs. 19.4%, *p* = 0.006).

**Conclusions:**

MII-pH monitoring effectively detected respiratory-related predictive parameters, including total/proximal reflux episodes and symptom correlations. We found that GERD patients with respiratory symptoms exhibited more proximal and total reflux episodes but not more acid-related episodes, as determined by MII-pH monitoring. Thus, such monitoring could be useful for diagnosing atypical GERD patients with respiratory symptoms. Furthermore, LTF exhibited a more significant effect on controlling typical symptoms in all GERD patients and reducing the recurrence rate than the Stretta procedure in patients with respiratory symptoms.

## Introduction

Gastroesophageal reflux disease (GERD) is defined as a condition that develops when the reflux of stomach contents causes troublesome symptoms and/or complications[1]. Typical symptoms of GERD include heartburn and regurgitation; however, GERD can also present with atypical symptoms that include other gastric and respiratory symptoms, such as non-cardiac chest pain, belching, cough, asthma, etc. In addition to financial burden[2], GERD also has a profound effect on the quality of life of affected individuals, especially patients with complaints of respiratory symptoms[3,4].

In recent years, 24-h ambulatory pH monitoring has been accepted as the gold standard for the diagnosis of GERD[5]. Recently, multichannel intraluminal impedance–pH monitoring (MII-pH) has been considered to be a more sensitive tool for diagnosing and characterizing the pathogenesis of GERD. This method can detect various types of esophageal reflux characteristics, including liquid, gas, acid, and nonacid characteristics[6–8].Thus far, studies have aimed to monitor abnormal MII-pH parameters or to evaluate the diagnostic usefulness of these parameters based on comparisons with pH monitoring[9,10]. Additionally, one study considered the diagnostic yield of MII-pH monitoring in patients undergoing proton pump inhibitor (PPI) therapy[11]. However, the effect of MII-pH monitoring on atypical GERD patients with respiratory symptoms has not yet been reported.

PPIs are solely anti-acid therapies that do not resolve the problem of non-erosive reflux disease [12] or esophageal motility abnormalities[13]. Moreover, up to 40% of GERD patients are refractory to PPIs[14,15]. In our previous study, we demonstrated that laparoscopic Toupet fundoplication (LTF) was more effective than the Stretta procedure in controlling GERD symptoms[16]. However, the effects of reflux on the upper respiratory tract, including chronic cough, asthma, expectoration, breathlessness and laryngospasm, seriously affect the quality of life of GERD patients [17–19]. Currently, no data regarding comparisons of patients with and without respiratory symptoms exist, and the efficiency of anti-reflux therapy (ART) in patients with respiratory symptoms remains to be assessed. Additionally, data concerning MII-pH in patients with respiratory symptoms remain lacking.

Therefore, in this study, we carefully re-analyzed data from previous GERD patients[16]. We grouped the patients by respiratory symptoms and prospectively assessed the diagnostic utility of MII-pH monitoring. Specifically, we compared the MII-pH parameters of patients with and without respiratory symptoms, and the results may reveal new clues for GERD patients with respiratory symptoms. Furthermore, we evaluated the 3-year outcomes of two different ART (LTF and Stretta procedures) in patients with respiratory symptoms (using patients with only gastrointestinal symptoms as controls) with the aim of assessing the diagnostic advantages of MII-pH and the efficiency of ART in controlling the recurrence of respiratory symptoms.

## Materials and Methods

### Ethics statement

This prospective observational study was approved by the Institutional Review Board at Xuanwu Hospital and the Second Artillery General Hospital of Chinese People’s Liberation Army and was conducted in compliance with the ethics principles for medical research involving human subjects as stated in the Declaration of Helsinki of the World Medical Association. All patients provided written informed consent.

### Subjects

All patients sought care in our department because standard medical treatment had produced no effects on their symptoms, which included respiratory and gastric symptoms. The inclusion criteria for the patients were the following: 1) GERD as diagnosed based on endoscopic evidence of esophagitis, 2) abnormal esophageal pH or impedance with correlated symptoms as recorded by MII-pH monitoring standards, 3) lower than normal esophageal sphincter (LES) pressure as detected by esophageal manometry, 4) non-responder to double-dose PPI therapy for over 8 weeks (less than 50% improvement in partial symptoms or no response with persistent symptoms, including typical and atypical symptoms), 5) no hiatal hernia or a small (<2 cm) hiatal hernia, and 6) age≥18 years. Patients with central nervous system diseases, other respiratory system diseases, connective tissue diseases, previous esophageal or gastric surgeries, esophageal strictures, shortened esophagi, impaired distal esophageal peristalsis, Barrett’s esophagus, autoimmune diseases, collagen vascular disease, and/or coagulation disorders were excluded.

The patients were asked to discontinue any medication that could influence esophageal motor function and gastric acid excretion (e.g., H_2_ receptor antagonists and PPIs) two weeks before the MII-pH monitoring. The gastric symptoms included heartburn, acid regurgitation, hiccups, belching and non-cardiac chest pain, and the respiratory symptoms included cough, expectoration, asthma, and shortness of breath. Only heartburn and acid regurgitation were considered typical GERD symptoms.

### MII-pH monitoring

The patients were required to fast overnight for at least 8 h before the MII-pH monitoring. The MII-pH probe consisted of a polyurethane catheter that included six impedance segments (each segment was 2 cm long) and one pH-measuring electrode (Sandhill Scientific, Highlands Ranch, CO, USA). The configuration of this catheter enabled the recording of changes in the intraluminal impedance at 3, 5, 7, 9, 15, and 17 cm above the LES. Additionally, the pH was monitored at 5 cm above the LES. The MII-pH probe was inserted transnasally, and the distal pH probe was positioned 5 cm above the LES as identified using high-resolution esophageal manometry. The data from the impedance channels and the pH electrodes were transmitted at a frequency of 50 Hz and stored on a portable data recorder (Sandhill Scientific, Highlands Ranch, CO, USA). The data recording was concluded after 24 h when the patients returned to the esophageal laboratory for probe removal. All data were uploaded onto a personal computer and analyzed using a commercially available software system (BioView Analysis; Sandhill Scientific Inc., Highlands Ranch, CO, USA). The patients were instructed to complete a diary that included indications of the beginning and ending times of meals and changes in body position and were asked to press an event marker button or posture button on a data logger whenever they experienced reflux symptoms or changed body position.

Acid exposure time (AET) was calculated as the percentage of time during which the pH was below 4 at the distal esophageal pH sensor, and AETs of 4.2% or greater were designated as abnormal thresholds. Additionally, a DeMeester score≥14.7 was also considered abnormal. The bolus exposure time (BET) consisted of the percentage of time that the refluxate was in contact with the distal esophageal impedance electrodes above the LES, and validated BET values of 1.4% or greater were considered abnormal. The records of the reflux episodes were designated as abnormal over 73 times, and proximal reflux episodes were considered when the refluxate reached the 15 cm impedance sensor (>15 cm, above the LES). The symptoms were considered related to reflux events if they occurred within 2 min after the reflux events. The symptom index (SI) and symptom association probability (SAP) were calculated and designated as positive when SI≥50% or SAP≥95%.

### Treatment

The GERD patients underwent one of two methods of ART, LTF or the endoscopic Stretta procedure, which is the standard-of-care for GERD patients[20,21], according to their own preferences. LTF was performed with five ports under general anesthesia. After dissecting the gastro-hepatic ligament with a harmonic scalpel, a window was created behind the lower esophagus. Then, the diaphragmatic crura were dissected carefully, and the distal esophagus was mobilized by approximately 5 cm, while the mediastinal structures, including the pleura, pericardium, vagus nerves and aorta, were identified and preserved. In all cases, the gastric fundus was dissected by dividing the short gastric vessels. The diaphragmatic crura were sewn behind the esophagus with 1–2 non-absorbable sutures, and a posterior 270° with a 2-cm-long fundoplication was constructed with 5–6 interrupted non-absorbable stitches.

Endoscopic Stretta procedures were performed on all patients as previously described[22,23]. Briefly, the patient was sedated, and the distance to the gastroesophageal junction was measured with a gastroscope. Then, the endoscope was withdrawn, and a radiofrequency-delivering catheter that consisted of a flexible balloon-basket with four electrode needle sheaths was introduced orally using a guide wire. The balloon was inflated 2 cm proximal to the squamo-columnar junction, the electrode needles were deployed, and the radiofrequency energy was delivered for 1 min. The needles were then withdrawn, the balloon was deflated, and the catheter was rotated bh 45°. These steps were serially repeated every 0.5 cm inwards to cover an area 2 cm above and 0.5 cm below the squamo-columnar junction.

### Outcome assessment

The aim was to evaluate the ART efficacies and compare these efficacies in the treatment of GERD patients with respiratory symptoms. The primary outcome measure of this study was the reflux symptom score: the frequency and severity of major GERD symptoms, including heartburn, regurgitation, non-cardiac chest pain, belching, hiccups, cough, expectoration, asthma and shortness of breath. The data related to these outcome measures were collected via a standardized questionnaire as previously described[22,24]. More specifically, the frequencies were graded as 0 (none), 1 (less than once per week), 2 (once or twice per week), 3 (three or four times per week), 4 (five or six times per week) and 5 (more than six times per week). The severities were graded as 0 (none), 1 (slight), 2 (mild), 3 (moderate), 4 (severe) and 5 (extremely severe). The total of the frequency score and the severity score for each of these measures was designated as the symptom score. Other outcome measures included medication independence, related complications and satisfaction with the treatment (not all/ partially/fully).

The questionnaires were prepared in simplified Chinese and administered to the subjects before the LTF or Stretta procedures and at 1 year and 3 years post-treatment.

### Statistical analysis

The data are expressed as the means ± the standard deviations (SDs) unless otherwise specified. The data were analyzed with Student’s t tests or nonparametric tests based on their nature. The SPSS-17.0 statistical analysis software (SPSS Inc., Chicago, IL, USA) was used. Differences were considered significant at *p*<0.05.

## Results

### Characteristics of the participants

**Fig 1:**
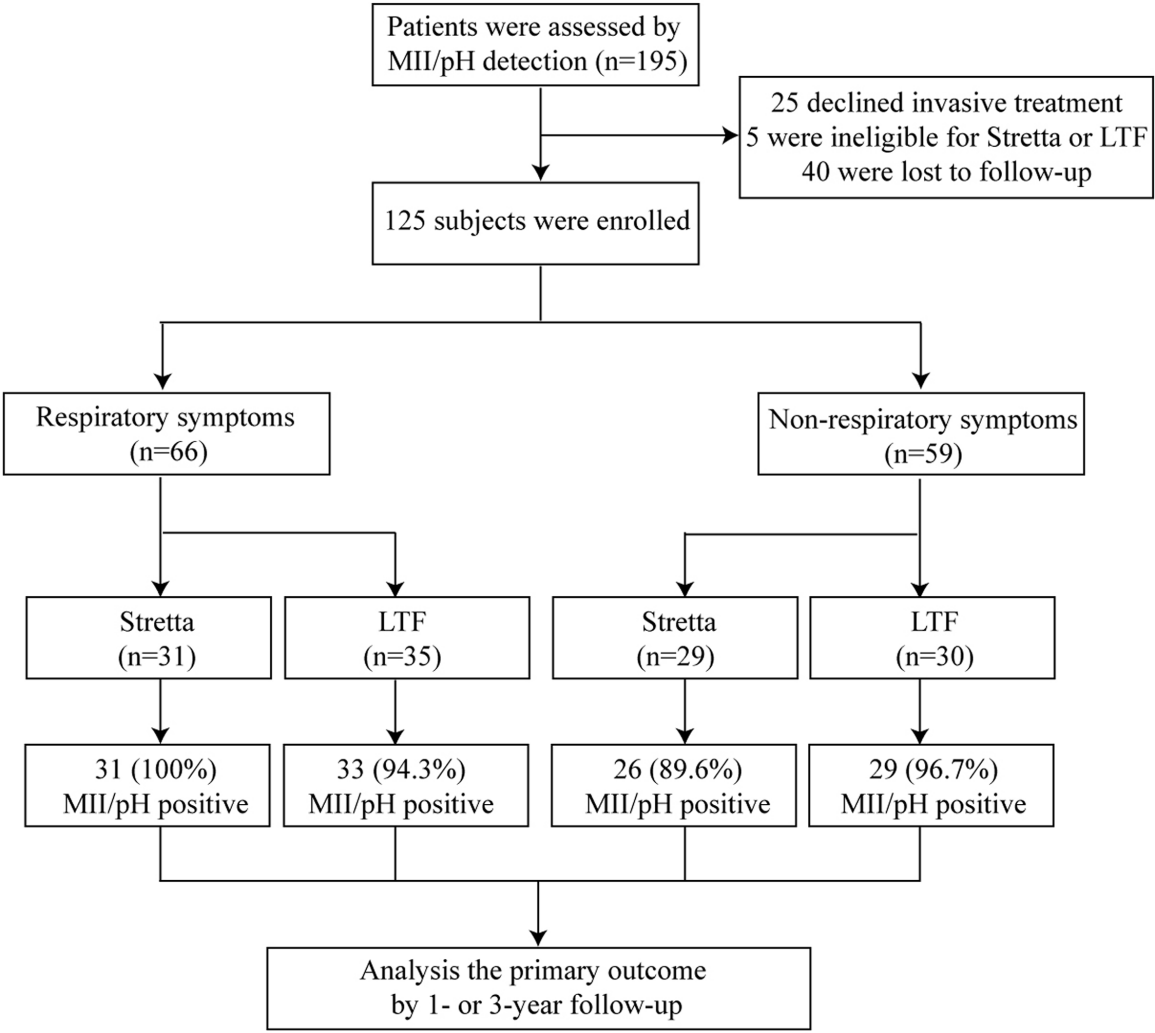
Enrollment, group, and follow-up of the study participants. All patients for whom diagnostic and follow-up data were available were included in the analysis regardless of whether they discontinued treatment.

Two hundred twelve patients with refractory GERD who sought care in our department were recruited between January 2011 and January 2012. Due to intolerance of transnasal intubation, the data from 17 patients were incomplete and were not included in the analysis. Of the remaining 195 patients, 96 (49.2%) had respiratory symptoms. Based on the definitions of abnormal MII-pH thresholds, 75 patients exhibited increased pH parameters, and 142 exhibited abnormal MII parameters. Moreover, 156 patients exhibited positive symptom correlations. Due to declines to undergo invasive ART (25 patients) and diagnoses of GERD with severe pulmonary fibrosis (5 patients), the data from 30 patients were only included in the MII-pH results and excluded from the outcome assessments. During the designated 3-year follow-up period, 40 patients dropped out of the study and were only included in the MII-pH results. Consequently, the remaining 125 (125/165, 75.8%) patients completed the follow-up, and the data from these patients comprised the ART (age, 47.8+12.3 y; 48.7% male) results. Of these patients, 60 (60/85, 70.6%) receiving the Stretta procedure and 65 (65/80, 81.3%) receiving LTF were included in the final analysis (Fig 1). Table 1 summarizes the characteristics of the enrolled subjects, and there are no significant differences in general characteristics, such as the gender ratio, age, and BMI, between patients with respiratory symptoms and those without (Table 2).

**Table 1:** Baseline Demographics, Clinical Characteristics, and Proportions with Abnormal pH and Impedance Parameters.

Characteristic	Total subjects(n = 195)	%
Age (y)	47.8±12.3	
Male	95	48.7
GERD Course (y)	13.1±10.3	
Smoking	32	16.4
Alcohol	78	40.0
BMI (kg/m^2^)	27.8±5.9	
Abnormal by MII/pH		
GERD by pH	75	38.5
GERD by MII	142	72.8
Symptom correlation	156	80.0
Typical symptoms		
Heartburn	145	74.4
Acid regurgitation	141	72.3
Atypical symptoms		
NCCP	54	27.7
Belching	75	38.5
Hiccup	24	12.3
Cough	71	36.4
Expectoration	39	20.0
Asthma	59	30.3
Short of breath	40	20.5
Invasive treatment	165	84.6
LTF	65/80*	81.3
Stretta	60/85*	70.6

Note. Values are given as mean ± SD or n (%). GERD = gastroesophageal reflux disease, MII = multichannel intraluminal impedance, NCCP = non-cardiac chest pain, LTF = laparoscopic Toupet fundoplication, SD = standard deviation.

*a/b: a represents patient with 3-year follow-up, b represents patient with LTF or Stretta procedure.

**Table 2:** Comparison of MII-pH and Manometry Data between the Patients with Respiratory Symptoms and Patients without Respiratory Symptoms.

Parameters	All subjects		Respiratory symptoms		Non-respiratory symptoms		*p*Value
	N	%	n	%	n	%	
Total	195	100.0	96	49.2	99	50.8	0.975
Age (y)	47.8±12.3		47.2±11.4		48.5±11.6		0.252
Male	95	48.7	45	46.9	50	50.5	0.467
BMI (kg/m^2^)	27.8±5.9		28.1±6.4		27.4±5.2		0.533
pH probe							
AET ≥4.2%	72	36.9	37	51.4	35	48.6	0.991
DeMesster≥14.72	68	34.9	34	50.0	34	50.0	0.840
Impedance probes							
BET≥1.4%	135	69.2	70	51.9	65	48.1	0.248
Reflux episode≥73	88	45.1	50	56.8	38	43.2	0.102
Total reflux episodes	67.6±27.0		70.7±29.3		64.7±24.4		**0.044**
Proximalreflux episodes	31.7±19.9		35.2±21.3		28.3±17.9		**0.013**
SAP	121	62.1	63	52.1	58	47.9	0.784
SI	137	70.3	68	49.6	69	50.4	0.904
Multiple positive							
AET+SAP	58	29.7	33	56.9	25	43.1	0.318
AET+SI	59	30.3	32	54.2	27	45.8	0.275
BET+SAP	100	51.3	56	56.0	44	44.0	**0.013**
BET+SI	114	58.5	58	50.9	56	49.1	0.147
Reflux episode+SAP	60	30.8	38	63.3	22	36.7	**0.029**
Reflux episode+SI	70	35.9	39	55.7	31	44.3	**0.049**
High-resolution manometry							
LES pressure (mm Hg)	14.7±4.8		12.7±4.0		15.8±5.7		**0.036**
UES pressure (mm Hg)	67.0±12.6		66.5±13.2		67.3±11.9		0.771
Dysmotility, n	19	9.7	11	0.8	8	11.1	0.516
Hiatus Hernia, n	29	14.9	13	13.5	16	16.1	0.614

Note. Values are given as the means ± SD or n (%). Bolded entries represent significant p values. AET = acid exposure time, BET = bolus exposure time, SAP = symptom association probability, SI = symptom index, UES = upper esophageal sphincter, SD = standard deviation

### Abnormal MII-pH parameters

In the total cohort, at least one item exceeded the MII-pH normal value, including the esophageal pH parameters, impedance parameters or symptom correlations and endoscopic esophagitis. Seventy-two (36.9%) participants had abnormal total AET values, and 68 (34.9%) exhibited DeMeester scores indicating pH detection. Regarding the impedance detection, 135 (69.2%) participants had abnormal total BETs, and 88 (45.1%) had abnormal episodes of reflux. The total reflux episodes were 67.6±27.0, and the proximal reflux episodes (>15 cm above the LES) were 31.7±19.9. Overall, reflux evidence with positive symptom correlations was demonstrated in 121 (62.1%) of the SAP and 137 (70.3%) of the SI participants (Table 2). In 75.4% of the patients, the SI and SAP were consistent, and the percentage of patients who were positive for both SI and SAP was the highest.

**Fig 2:**
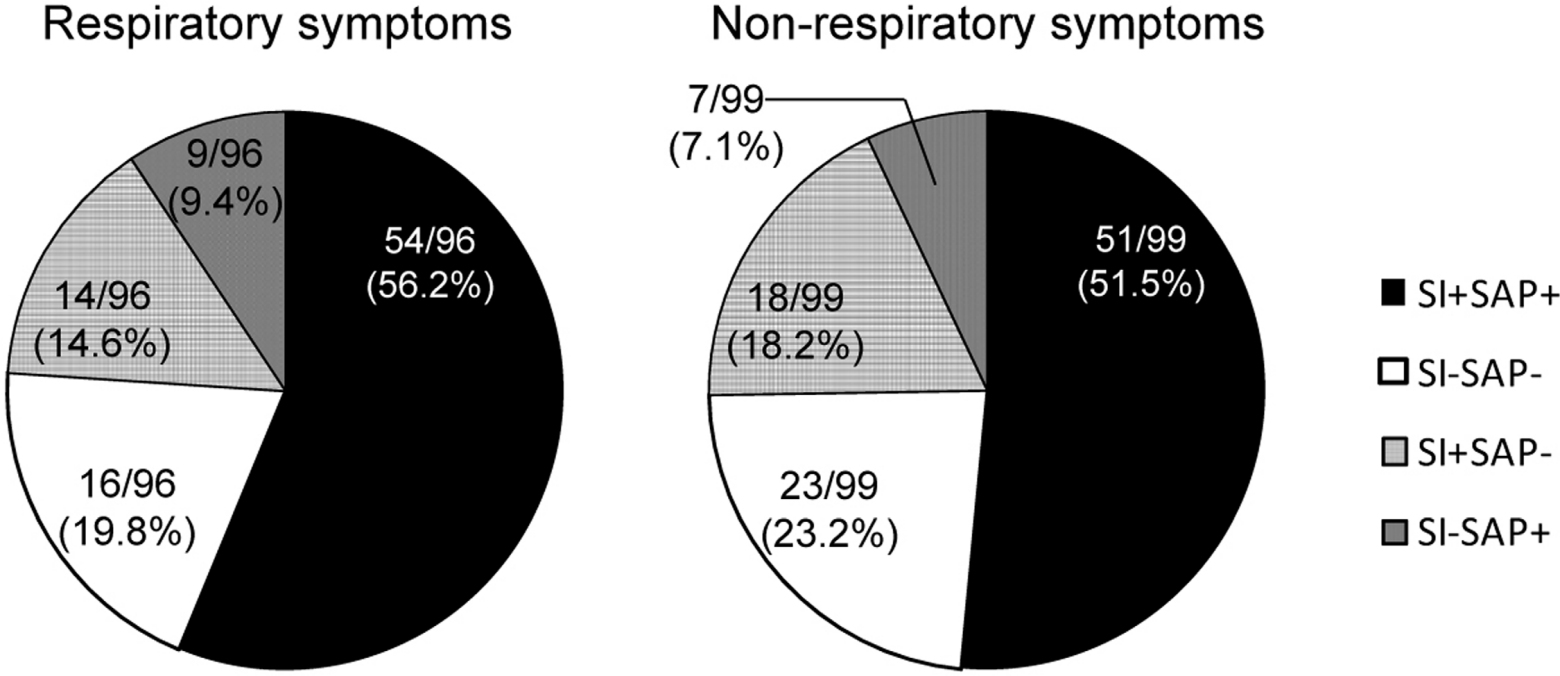
Positive distribution and consistency of SI and SAP of the respiratory symptoms and non-respiratory symptoms.

Next, the patients were classified into respiratory and non-respiratory symptom groups according to their complaints of respiratory symptoms. There were no significant differences in age or gender between the groups (respiratory symptoms: age, 47.2±11.4 y, 46.9% male; non-respiratory symptoms: age, 48.5±11.6 y, 50.5% male). The respiratory symptoms group exhibited significantly more reflux episodes and proximal reflux episodes than the non-respiratory symptoms group (Table 2). More precisely, with multiple analyses, the BET+SAP and reflux episodes+SI/SAP were also found to occur more frequently in the respiratory symptoms group than in the non-respiratory symptoms group. However, both groups showed similar rates of abnormal AET, BET, DeMeester scores and positivity for SI and SAP (Table 2 and Fig 2). Among the manometric examination, only lower LES pressure was found in patients with respiratory symptoms, for which there was a significant difference between the two groups (respiratory symptoms: 12.7±4.0 mmHg, non-respiratory symptoms: 15.8±5.7 mmHg, *p* = 0.036) (Table 2).

### ART efficacy in GERD patients with respiratory symptoms

We performed a prespecified subgroup analysis according to whether patients received LTF or the Stretta procedure to determine the ART outcomes while controlling for respiratory symptoms that were likely to benefit from MII-pH monitoring diagnoses. Moreover, we also compared the ART efficacy and persistence in these participants to those in the patients with only gastrointestinal symptoms. During the 1- and 3-year follow-ups, both the LTF and Stretta procedures effectively reduced the respiratory and gastrointestinal symptom scores of the patients with MII-pH diagnoses (Tables 3 and 4). Although there was no significant difference in the pre-treatment symptom scores between the patients who underwent LTF and those who underwent Stretta (Table 3), both procedures exhibited similar effects on respiratory symptoms, including cough, expectoration, asthma and shortness of breath, and these benefits were sustained for 3 years; however, LTF clearly resulted in significant benefits according to a reduced re-operation rate and higher satisfaction rate than Stretta (Table 4). Moreover, LTF had a better effect on improving heartburn, regurgitation and non-cardiac chest pain in the patients with respiratory symptoms (Table 4) and the control group patients with gastrointestinal symptoms only (S1 and S2 Tables).The patients who underwent LTF surgery did not exhibit benefits in terms of PPI independence or related complications (surgery-related abdominal distension) at the end of the 3-year follow-up. Furthermore, 8 patients who were excluded from the final symptom score analysis underwent revision surgeries in the 3-year follow-up. Among these 8 patients, 6 had respiratory symptoms, and we found a clearly increased recurrence rate among those who underwent the Stretta procedure compared with those who underwent LTF in the 3-year follow-up analysis of outcomes (Table 4).

**Table 3:** Comparison of Clinical Characteristics in Respiratory Symptoms Patients prior to Stretta and LTF.

Characteristics	Stretta(n = 31)		LTF(n = 35)		*p*Value
Age (y)	47.7±10.2		48.4±12.5		0.807
Male	12(38.7%)		17(48.6%)		0.428
Symptom score^a^					
Acid regurgitation^b^	7.20±0.86	15/31	7.68±0.65	22/35	0.078
Heartburn^b^	7.27±0.80	15/31	7.61±0.78	23/35	0.203
NCCP	7.25±0.50	4/31	7.09±0.83	11/35	0.728
Belching	7.15±0.69	13/31	7.00±0.63	6/35	0.367
Hiccup	6.33±0.58	3/31	7.00±0.00	2/35	0.219
Cough	7.70±0.57	20/31	7.69±0.62	26/35	0.380
Expectoration	7.47±0.70	19/31	7.30±0.92	20/35	0.456
Asthma	7.80±0.68	15/31	7.52±0.85	27/35	0.205
Short of breath	7.54±0.88	13/31	7.44±0.73	16/35	0.101

Note. Values are given as the means ± SD or n(%). NCCP = non-cardiac chest pain, LTF = laparoscopic Toupet fundoplication, SD = standard deviation.

a The total of the frequency score and the severity score for each symptom was designated as the symptom score.

b GERD typical symptoms.

**Table 4:** Post-treatment Outcomes in Respiratory Symptoms Patients between Stretta and LTF procedure.

Characteristics	1-Year Follow-Up			3- Year Follow-Up		
	Stretta	LTF	*p* Value	Stretta	LTF	*p* Value
Symptom score ^a^						
Acid regurgitation ^b^	2.93±2.37	1.36±2.19	**0.046**	3.73±2.25	1.96±2.45	**0.029**
Heartburn ^b^	2.73±2.68	1.61±2.55	0.202	3.80±2.68	1.95±2.40	**0.040**
NCCP	5.25±2.22	1.36±1.12	**0.007**	6.25±1.70	2.27±2.24	**0.007**
Belching	3.23±2.49	2.64±1.96	0.528	4.08±2.10	3.73±2.64	0.722
Hiccup	2.33±0.58	3.50±2.12	0.402	2.33±0.58	3.50±2.12	0.402
Cough	4.20±2.58	4.36±2.68	0.841	4.60±2.52	4.80±2.36	0.785
Expectoration	3.95±2.32	3.95±2.74	0.995	4.36±2.29	4.74±2.35	0.280
Asthma	3.80±2.57	4.12±2.69	0.715	4.20±2.62	4.65±2.62	0.597
Short of breath	3.84±2.08	3.50±2.90	0.721	4.31±1.97	4.50±2.85	0.841
PPI use, n	8	10	0.805	9	10	0.968
Complication, n						
Abdominal distension	0	2	0.182	0	2	0.182
Re-operation, n	1	0	0.291	6	0	**0.006**
Satisfaction^c^, n	25	30	0.591	20	29	**0.046**

Note. Values are given as the means ± SD or n. Bolded entries represent significant p values. NCCP = non-cardiac chest pain, LTF = laparoscopic Toupet fundoplication, PPI = proton pump inhibitors, SD = standard deviation.

a The total of the frequency score and the severity score for each symptom was designated as the symptom score.

b GERD typical symptoms.

c Satisfaction is counted as fully or partially satisfied with the treatment.

## Discussion

Combined MII-pH monitoring is considered to be the most sensitive tool for assessing all types of gastroesophageal reflux events (i.e., acidic, weakly acidic and weakly alkaline events), their composition, proximal extent, duration and clearing[6,25]. In this study, 195 patients diagnosed with GERD by MII-pH monitoring were enrolled. Only 75 of these patients exhibited positive findings by pH monitoring; however, 142 patients exceeded the upper limits of the normal MII parameters, and 156 exhibited positive symptom correlations. These findings revealed the diagnostic utility of MII-pH monitoring in patients with suspected GERD. Traditional pH parameters have a well-established predictive value in GERD, and patients with abnormal pH parameters can benefit from anti-secretory therapy[26] and ART[27]. However, outcome data regarding impedance parameters and follow-up data for ART are lacking in the literature despite the increased diagnostic yield of MII-pH testing over pH testing alone. We introduced multiple impedance parameters, including BET, total and proximal reflux episodes, SI and SAP, which can prevent false-negative GERD findings in patients. Moreover, to prevent false-positives, all of the thresholds of the MII parameters were designed according to existing studies[8,28,29]. A comparison of the pre- and post-LTF and Stretta procedure outcomes revealed that all of the typical and atypical symptoms of GERD were improved regardless of the presence of gastric or respiratory symptoms.

Because the respiratory and gastric systems share common channels with the oropharynx, some atypical symptoms are manifested as respiratory symptoms, which appear more serious than gastric symptoms. Interestingly, we found that 5 patients with diagnoses of pulmonary fibrosis were unable to tolerate any anesthesia and exhibited poor lung function. A recent article documented causal links of GERD with asthma, chronic cough and other lung diseases. The underlying mechanism of respiratory generation in GERD is due to the hypo-pressure of LES and excessive transient LES relaxation and by the stomach contents directly refluxing from the distal esophagus to the proximal esophagus with or without esophageal dysmotility, which could form a spray according to the special structure of the laryngopharynx. The spray induces micro-aspirations or macro-aspirations into the upper respiratory tract, resulting in irritability and symptoms such as cough, expectoration, asthma and others [30–32]. In this study, the GERD patients with respiratory symptoms were isolated to analyze the MII-pH results and follow-up outcomes following invasive treatment. We demonstrated that the subjects with respiratory symptoms exhibited more reflux episodes than those without respiratory symptoms, especially proximal reflux episodes (>15 cm above LES). These results indicate that proximal reflux episodes could be a common cause of respiratory symptoms. Moreover, the respiratory symptoms caused by GERD maybe direct effects of upper airway injuries caused by gastric contents refluxing above the upper esophageal sphincter (proximal reflux episodes) and aspiration into the bronchial tree; our center established this mechanism using a special pharyngeal nozzle structure[33,34]. Interestingly, total reflux episodes also contributed to the increase in the respiratory symptom incidence rate shown in our results. This increase may be related to the reflexive vagally mediated airway responses mechanisms that occur during reflux events and are limited to the lower esophagus. Additionally, esophageal motility abnormalities may occur with GERD because this study revealed a high rate of abnormal BETs by MII-pH and dysmotility by manometry. Although the frequency of BET-positive cases with SAP conditions was greater among the respiratory patients in our study, the relationship between the respiratory symptoms of GERD and esophageal motility abnormalities requires further study. Overall, the mechanism of respiratory symptom generation is complicated including the lower pressure of LES, proximal reflux, pharyngolarynx spray, vagally mediated airway responses and esophageal dysmotility, abnormal reflux scores by MII-pH monitoring just an important factor contributed to the respiratory symptoms generation, this does not follow “all-or-none” law.

Laparoscopic fundoplication is considered the gold standard surgical treatment for GERD and is administered via two methods, i.e., the Nissen and Toupet methods[35,36]. Laparoscopic Toupet fundoplication has the benefit of reducing postoperative dysphagia and has thus become a widely used surgical treatment for GERD[37]. Recently, the minimally invasive Stretta procedure has become another effective option for patients who are PPI-refractory and poor surgical candidates but still require intensive treatment to adequately manage their GERD[22,23]. Our results clearly demonstrate that both of these procedures effectively reduce the frequency and severity of GERD-associated symptoms, including typical and atypical symptoms, and indirectly prove the accuracy of MII-pH monitoring. Specifically, based on our subgroup analyses, the LTF and Stretta procedures equally controlled the patients’ respiratory symptoms; LTF achieved greater control of the typical symptoms and non-cardiac chest pain compared with the Stretta procedure regardless of the combinations of respiratory symptoms. However, controversy remains regarding the use of laparoscopic fundoplication to control GERD-related respiratory symptoms[38,39]. Although there was no significant difference in the control of respiratory symptoms between the LTF and Stretta procedures, the rate of re-operation following the Stretta procedure was 3.2% over the 1-year follow-up, and this rate increased to 19.4% at the end of the 3-year follow-up only in the respiratory symptoms group, which indicates that patients with respiratory symptoms may need to prudently select the fundoplication method to achieve a long-term effect. Despite the benefits in terms of repeatability, the wider application of the Stretta procedure will be limited by the associated increased recurrence rate. Notably, for elderly patients, Stretta was the only procedure to relieve symptoms with minimal risks related to anesthesia and surgery.

Unfortunately, there were some limitations to our study that should be acknowledged. First, MII-pH monitoring is a costly and time-consuming technique that is still not widely available for use in follow-up, and additional objective results are lacking regarding evaluating the effect of ART. Second, despite the improved diagnostic yield from MII-pH, more specific diagnoses for the detection of laryngo-pharyngeal reflux remain to be determined. Third, the sample size should be enlarged, and the patient enrollment and trial design should be more prospective and random.

## Conclusions

Multichannel intraluminal impedance-pH monitoring effectively detected respiratory-related predictive parameters, including total/proximal reflux episodes and symptom correlations. We found that GERD patients with respiratory symptoms exhibited more proximal and total reflux episodes but not more acid-related episodes, as determined by multichannel intraluminal impedance-pH monitoring and lower pressure of lower esophageal sphincter by manometry. Thus, such monitoring could be useful for diagnosing atypical GERD patients with respiratory symptoms. Furthermore, laparoscopic Toupet fundoplication exhibited a more significant effect on controlling typical symptoms in all GERD patients and reducing the recurrence rate than the Stretta procedure in patients with respiratory symptoms.

## Supporting Information

S1 Table Comparsion of Clinical Characteristics in Non-respiratory Symptoms prior to Stretta and LTF. (DOCX)

S2 Table Post-treatment Outcomes in Non-respiratory Symptoms Patients between Stretta and LTF procedure. (DOCX)

## Abbreviations

GERD: gastroesophageal reflux disease
MII-pH: multichannel intraluminal impedance-pH
PPI: proton pump inhibitors
ART: anti-reflux therapy
LTF: laparoscopic Toupet fundoplication
AET: acid exposure time
BET: bolus exposure time
SAP: symptom association probability
SI: symptom index
LES: lower esophageal sphincter

## References

[1] Vakil N, van Zanten SV, Kahrilas P, Dent J, Jones R. The Montreal definition and classification of gastroesophageal reflux disease: a global evidence-based consensus. Am J Gastroenterol. 2006;101: 1900–1920; quiz 1943. 10.1111/j.1572-0241.2006.00630.x 16928254

[2] Comay D, Adam V, da Silveira EB, Kennedy W, Mayrand S, Barkun AN. The Stretta procedure versus proton pump inhibitors and laparoscopic Nissen fundoplication in the management of gastroesophageal reflux disease: a cost-effectiveness analysis. Can J Gastroenterol. 2008;22: 552–558. 18560633 10.1155/2008/765458 PMC2660813

[3] Wu JC. Gastroesophageal reflux disease: an Asian perspective. J Gastroenterol Hepatol. 2008;23: 1785–1793. 10.1111/j.1440-1746.2008.05684.x 19120871

[4] Bruley des Varannes S, Coron E, Galmiche JP. Short and long-term PPI treatment for GERD. Do we need more-potent anti-secretory drugs? Best Pract Res Clin Gastroenterol. 2010;24: 905–921. 10.1016/j.bpg.2010.09.004 21126703

[5] Madan K, Ahuja V, Gupta SD, Bal C, Kapoor A, Sharma MP. Impact of 24-h esophageal pH monitoring on the diagnosis of gastroesophageal reflux disease: defining the gold standard. J Gastroenterol Hepatol. 2005;20: 30–37. 10.1111/j.1440-1746.2004.03530.x 15610443

[6] Tutuian R, Castell DO. Gastroesophageal reflux monitoring: pH and impedance. GI Motility Online. 2006;5 10.1038/gimo31

[7] Lee BE, Kim GH, Ryu DY, Kim DU, Cheong JH, Lee DG, et al Combined dual channel impedance/pH-metry in patients with suspected laryngopharyngeal reflux. J Neurogastroenterol Motil. 2010;16: 157–165. 10.5056/jnm.2010.16.2.157 20535346 PMC2879840

[8] Khan MQ, Alaraj A, Alsohaibani F, Al-Kahtani K, Jbarah S, Al-Ashgar H. Diagnostic utility of impedance-pH monitoring in refractory non-erosive reflux disease. J Neurogastroenterol Motil. 2014;20: 497–505. 10.5056/jnm14038 25273120 PMC4204403

[9] Rosen R, Hart K, Nurko S. Does reflux monitoring with multichannel intraluminal impedance change clinical decision making? J Pediatr Gastroenterol Nutr. 2011;52: 404–407. 10.1097/MPG.0b013e3182078081 21407105 PMC3293158

[10] Loots CM, Benninga MA, Davidson GP, Omari TI. Addition of pH-impedance monitoring to standard pH monitoring increases the yield of symptom association analysis in infants and children with gastroesophageal reflux. J Pediatr. 2009;154: 248–252. 10.1016/j.jpeds.2008.08.019 18823910

[11] Zerbib F, Roman S, Ropert A, des Varannes SB, Pouderoux P, Chaput U, et al Esophageal pH-impedance monitoring and symptom analysis in GERD: a study in patients off and on therapy. Am J Gastroenterol. 2006;101: 1956–1963. 10.1111/j.1572-0241.2006.00711.x 16848801

[12] Savarino E, Zentilin P, Tutuian R, Pohl D, Casa DD, Frazzoni M, et al The role of nonacid reflux in NERD: lessons learned from impedance-pH monitoring in 150 patients off therapy. Am J Gastroenterol. 2008;103: 2685–2693. 10.1111/j.1572-0241.2008.02119.x 18775017

[13] Blonski W, Hila A, Vela MF, Castell DO. An analysis of distal esophageal impedance in individuals with and without esophageal motility abnormalities. J Clin Gastroenterol. 2008;42: 776–781. 10.1097/MCG.0b013e31806daf77 18360293

[14] Moraes-Filho JP. Refractory gastroesophageal reflux disease. Arq Gastroenterol. 2012;49: 296–301. 10.1590/S0004-28032012000400012 23329226

[15] Cicala M, Emerenziani S, Guarino MP, Ribolsi M. Proton pump inhibitor resistance, the real challenge in gastro-esophageal reflux disease. World J Gastroenterol. 2013;19: 6529–6535. 10.3748/wjg.v19.i39.6529 24151377 PMC3801364

[16] Liang WT, Yan C, Wang ZG, Wu JM, Hu ZW, Zhan XL, et al Early and midterm outcome after laparoscopic fundoplication and a minimally invasive endoscopic procedure in patients with gastroesophageal reflux disease: a prospective observational study. J Laparoendosc Adv Surg Tech A. 2015;25: 657–661. 10.1089/lap.2015.0188 26258269

[17] Gislason T, Janson C, Vermeire P, Plaschke P, Bjornsson E, Gislason D, et al Respiratory symptoms and nocturnal gastroesophageal reflux: a population-based study of young adults in three European countries. Chest. 2002;121: 158–163. 10.1378/chest.121.1.158 11796445

[18] Lee JH, Park SY, Cho SB, Lee WS, Park CH, Koh YI, et al Reflux episode reaching the proximal esophagus are associated with chronic cough. Gut Liver. 2012;6: 197–202. 10.5009/gnl.2012.6.2.197 22570748 PMC3343157

[19] Katz PO, Gerson LB, Vela MF. Guidelines for the diagnosis and management of gastroesophageal reflux disease. Am J Gastroenterol. 2013;108: 308–328; quiz 329. 10.1038/ajg.2012.444 23419381

[20] Shaw JM, Bornman PC, Callanan MD, Beckingham IJ, Metz DC. Long-term outcome of laparoscopic Nissen and laparoscopic Toupet fundoplication for gastroesophageal reflux disease: a prospective, randomized trial. Surg Endosc. 2010;24: 924–932. 10.1007/s00464-009-0700-3 19789920

[21] Aziz AM, El-Khayat HR, Sadek A, Mattar SG, McNulty G, Kongkam P, et al A prospective randomized trial of sham, single-dose Stretta, and double-dose Stretta for the treatment of gastroesophageal reflux disease. Surg Endosc. 2010;24: 818–825. 10.1007/s00464-009-0671-4 19730952

[22] Liang WT, Wu JM, Wang F, Hu ZW, Wang ZG. Stretta radiofrequency for gastroesophageal reflux disease-related respiratory symptoms: a prospective 5-year study. Minerva Chir. 2014;69: 293–299. 25267020

[23] Liang WT, Wu JN, Wang F, Hu ZW, Wang ZG, Ji T, et al Five-year follow-up of a prospective study comparing laparoscopic Nissen fundoplication with Stretta radiofrequency for gastroesophageal reflux disease. Minerva Chir. 2014;69: 217–223. 24987969

[24] Liang WT, Wang ZG, Wang F, Yang Y, Hu ZW, Liu JJ, et al Long-term outcomes of patients with refractory gastroesophageal reflux disease following a minimally invasive endoscopic procedure: a prospective observational study. BMC Gastroenterol. 2014;14: 178 10.1186/1471-230X-14-178 25304252 PMC4287567

[25] Cho YK, Kim GH, Kim JH, Jung HY, Lee JS, Kim NY. [Diagnosis of gastroesophageal reflux disease: a systematic review]. Korean J Gastroenterol. 2010;55: 279–295. 10.4166/kjg.2010.55.5.279 20697188

[26] Aanen MC, Weusten BL, Numans ME, de Wit NJ, Samsom M, Smout AJ. Effect of proton-pump inhibitor treatment on symptoms and quality of life in GERD patients depends on the symptom-reflux association. J Clin Gastroenterol. 2008;42: 441–447. 10.1097/MCG.0b013e318074dd62 18344896

[27] Soyer T, Karnak I, Tanyel FC, Senocak ME, Ciftci AO, Buyukpamukcu N. The use of pH monitoring and esophageal manometry in the evaluation of results of surgical therapy for gastroesophageal reflux disease. Eur J Pediatr Surg. 2007;17: 158–162. 10.1055/s-2007-965393 17638153

[28] Kohata Y, Fujiwara Y, Machida H, Okazaki H, Yamagami H, Tanigawa T, et al Pathogenesis of proton-pump inhibitor-refractory non-erosive reflux disease according to multichannel intraluminal impedance-pH monitoring. J Gastroenterol Hepatol. 2012;27 Suppl 3: 58–62. 10.1111/j.1440-1746.2012.07074.x 22486873

[29] Patel A, Sayuk GS, Gyawali CP. Parameters on esophageal pH-impedance monitoring that predict outcomes of patients with gastroesophageal reflux disease. Clin Gastroenterol Hepatol. 2015;13: 884–891. 10.1016/j.cgh.2014.08.029 25158924 PMC4339660

[30] Napierkowski J, Wong RK. Extraesophageal manifestations of GERD. Am J Med Sci. 2003;326: 285–299. 14615670 10.1097/00000441-200311000-00005

[31] Morehead RS. Gastro-oesophageal reflux disease and non-asthma lung disease. Eur Respir Rev. 2009;18: 233–243. 10.1183/09059180.00002509 20956148

[32] de Bortoli N, Nacci A, Savarino E, Martinucci I, Bellini M, Fattori B, et al How many cases of laryngopharyngeal reflux suspected by laryngoscopy are gastroesophageal reflux disease-related? World J Gastroenterol. 2012;18: 4363–4370. 10.3748/wjg.v18.i32.4363 22969200 PMC3436052

[33] Zhu G, Wang Z, Hu Z, Gao X, Ji F, Liang W. Pharyngeal nozzle and its spray: gastroesophageal reflux indult on airway. Chin J Exp Surg (Zhonghua Shi Yan Wai Ke Za Zhi). 2014;02: 3330–3332.

[34] Wang Z, Hu Z, Wu J, Ji F, Wang H, Lai Y, et al Insult of gastroesophageal reflux on airway: clinical significance of pharyngeal nozzle. Front Med. 2015;9: 117–122. 10.1007/s11684-014-0343-1 25034240

[35] Rothenberg SS. Two decades of experience with laparoscopic nissen fundoplication in infants and children: a critical evaluation of indications, technique, and results. J Laparoendosc Adv Surg Tech A. 2013;23: 791–794. 10.1089/lap.2013.0299 23941587

[36] Kirschniak A, Pointner R, Granderath FA. [Laparoscopic Toupet—fundoplication]. Zentralbl Chir. 2013;138: 397–399. 10.1055/s-0033-1350636 23950075

[37] Qin M, Ding G, Yang H. A clinical comparison of laparoscopic Nissen and Toupet fundoplication for gastroesophageal reflux disease. J Laparoendosc Adv Surg Tech A. 2013;23: 601–604. 10.1089/lap.2012.0485 23614820

[38] Thoman DS, Hui TT, Spyrou M, Phillips EH. Laparoscopic antireflux surgery and its effect on cough in patients with gastroesophageal reflux disease. J Gastrointest Surg. 2002;6: 17–21. 11986013 10.1016/s1091-255x(01)00013-0

[39] Koch OO, Antoniou SA, Kaindlstorfer A, Asche KU, Granderath FA, Pointner R. Effectiveness of laparoscopic total and partial fundoplication on extraesophageal manifestations of gastroesophageal reflux disease: a randomized study. Surg Laparosc Endosc Percutan Tech. 2012;22: 387–391. 10.1097/SLE.0b013e31825efb5b 23047378

