# Supplementary material for: Diagnosis and Anti-Reflux Therapy for GERD with Respiratory Symptoms: A Study Using Multichannel Intraluminal Impedance-pH Monitoring
Source: PLoS One. 2016 Aug 17;11(8):e0160139. doi: 10.1371/journal.pone.0160139 (PMC4988652; doi:10.1371/journal.pone.0160139)
Supplement: S1 Table — (DOCX) [file pone.0160139.s001.docx]

| **S1 Table.** Comparsion of Clinical Characteristics in Non-respiratory Symptoms prior to Stretta and LTF | | | | | |
| --- | --- | --- | --- | --- | --- |
| **Characteristics** | **Stretta**  **(n=29)** | | **LTF**  **(n=30)** | | ***p* Value** |
| Age | 47.1±11.4 | | 49.3±11.7 | | 0.579 |
| Male | 13(44.8%) | | 18(60.0%) | | 0.251 |
| Symptom score^a^ |  |  |  |  |  |
| Acid regurgitation^b^ | 7.76±0.52 | 25/29 | 7.85±0.86 | 27/30 | 0.648 |
| Heartburn^b^ | 7.74±0.52 | 27/29 | 7.84±0.73 | 26/30 | 0.548 |
| NCCP | 7.56±0.73 | 9/29 | 7.78±0.70 | 14/30 | 0.456 |
| Belching | 7.06±0.83 | 17/29 | 7.60±0.52 | 10/30 | 0.075 |
| Hiccup | 7.00±0.89 | 6/29 | 7.50±0.58 | 4/30 | 0.314 |
| Note. Values are given as the means ± SD or n(%).  NCCP= non-cardiac chest pain, LTF=laparoscopic Toupet fundoplication, SD=standard deviation.  ^a^ The total of the frequency score and the severity score for each symptom was designated as the symptom score.  ^b^ GERD typical symptoms. | | | | | |
