# Supplementary material for: Diagnosis and Anti-Reflux Therapy for GERD with Respiratory Symptoms: A Study Using Multichannel Intraluminal Impedance-pH Monitoring
Source: PLoS One. 2016 Aug 17;11(8):e0160139. doi: 10.1371/journal.pone.0160139 (PMC4988652; doi:10.1371/journal.pone.0160139)
Supplement: S2 Table — (DOCX) [file pone.0160139.s002.docx]

| **S2 Table.** Post-treatment Outcomes in Non-respiratory Symptoms Patients between Stretta and LTF procedure | | | | | | |
| --- | --- | --- | --- | --- | --- | --- |
| **Characteristics** | **1-Year Follow-Up** | | | **3- Year Follow-Up** | | |
|  | **Stretta** | **LTF** | ***p* Value** | **Stretta** | **LTF** | ***p* Value** |
| Symptom score ^a^ |  |  |  |  |  |  |
| Acid regurgitation ^b^ | 3.52±3.00 | 1.96±2.42 | **0.044** | 4.20±2.86 | 2.67±2.27 | **0.036** |
| Heartburn ^b^ | 3.56±2.94 | 2.00±2.05 | **0.031** | 4.18±2.80 | 2.78±2.26 | **0.047** |
| NCCP | 4.67±2.78 | 2.86±2.85 | 0.149 | 5.22±2.54 | 3.00±2.27 | **0.037** |
| Belching | 3.06±2.86 | 2.45±2.50 | 0.546 | 3.41±2.76 | 2.90±2.66 | 0.638 |
| Hiccup | 2.17±2.23 | 4.00±3.36 | 0.326 | 2.67±2.73 | 4.25±3.30 | 0.431 |
| PPI use, n(%) | 9 | 7 | 0.514 | 10 | 8 | 0.523 |
| Complication, n(%) |  |  |  |  |  |  |
| Abdominal distension | 0 | 2 | 0.163 | 0 | 2 | 0.163 |
| Re-operation, n(%) | 0 | 0 | 1.000 | 2 | 0 | 0.148 |
| Satisfaction^c^, n | 25 | 28 | 0.627 | 23 | 27 | 0.427 |
| Note. Values are given as the means ± SD or n. Bolded entries represent significant *p* values.  NCCP= non-cardiac chest pain, LTF=laparoscopic Toupet fundoplication, PPI= proton pump inhibitors , SD=standard deviation.  ^a^ The total of the frequency score and the severity score for each symptom was designated as the symptom score.  ^b^ GERD typical symptoms.  ^c^ Satisfaction is counted by fully or partially satisfied with the treatment. | | | | | | |
